# Methyl-Beta-Cyclodextrin-Induced Macropinocytosis Results in Increased Infection of Sf21 Cells by Bombyx Mori Nucleopolyhedrovirus

**DOI:** 10.3390/v11100937

**Published:** 2019-10-11

**Authors:** Jinshan Huang, Chenya Li, Fengxiu Fan, Na Liu, Frank Boadi, Xingjia Shen, Bifang Hao

**Affiliations:** 1 Jiangsu Key Laboratory of Sericultural Biology and Biotechnology, School of Biotechnology, Jiangsu University of Science and Technology, Zhenjiang 212018, Jiangsu, China; jshuang@just.edu.cn (J.H.); lichenya0514@163.com (C.L.); fengxiu1216@126.com (F.F.); 13793161442@163.com (N.L.); frakboadi@gmail.com (F.B.); shenxjsri@163.com (X.S.); 2 Key Laboratory of Genetic Improvement of Sericulture in the Ministry of Agriculture, Sericultural Research Institute, Chinese Academy of Agricultural Science, Zhenjiang 212018, Jiangsu, China

**Keywords:** baculovirus, endocytosis, entry, host range, methyl-beta-cyclodextrin

## Abstract

Bombyx mori nucleopolyhedrovirus (BmNPV) is closely related to Autographa californica multiple nucleopolyhedrovirus (AcMNPV) with over ~93% amino acid sequence identity. However, their host ranges are essentially nonoverlapping. The mechanism of BmNPV entry into host cells is completely different from that of AcMNPV, and whether the entry mechanism difference relates to the host range remains unclear. BmNPV produces an abortive infection in nonhost cells due to virion nuclear transportation failure. Here, we performed a detailed study by increasing BmNPV infection in Sf21 cells with the aid of methyl-beta-cyclodextrin (MβCD). We found that low-concentration MβCD incubation efficiently activates membrane ruffling in Sf21 cells, which mediates the increase in BmNPV infection. Interestingly, MβCD incubation after virion internalization also increases the infection, which suggests that macropinocytosis is involved in BmNPV infection in Sf21 cells after virion internalization. Further study revealed that clathrin-mediated endocytosis (CME) is employed by BmNPV to facilitate entry into Sf21 cells, and chlorpromazine application abolishes BmNPV infection in cells incubated both with and without MβCD. Based on these studies, we show that BmNPV enters Sf21 cells via CME and that parallel induction of macropinocytosis facilitates BmNPV infection in Sf21 cells. This study reveals the mechanism of BmNPV entry into Sf21 cells and provides clues for improving BmNPV infections in nonpermissive cells.

## 1. Introduction

Baculoviruses, which have important applications in the areas of insect pesticides, protein expression, and gene therapy, are pathogens that infect insects from the orders Lepidoptera, Diptera, and Hymenoptera [1]. Each baculovirus exhibits a unique host specificity [2] where a virus can only infect a specific insect or a limited number of insects in the same order. The two insect alphabaculoviruses, Bombyx mori nucleopolyhedrovirus (BmNPV) and Autographa californica multiple nucleopolyhedrovirus (AcMNPV), are closely related to homologous ORFs, showing an ~93% amino acid sequence identity [3], but exhibit nonoverlapping host specificity. AcMNPV infects a much more diverse set of insects and insect cell lines than BmNPV [4]. For example, AcMNPV replicates in Spodoptera frugiperda (Sf) cells, but not in Bombyx mori (BmN) cells; conversely, BmNPV replicates in BmN cells, but not Sf cells, and almost all of the BmNPV gene expression in Sf9 cells is greatly reduced [5]. Thus far, several genes such as *dna helicase* [6,7,8,9] and *gp64* [1,4,10] have been suggested as determinants of the BmNPV and AcMNPV host range, but the mechanism is still unclear [1].

The endocytic entry of viruses occurs in a stepwise manner and is involved in virus binding, signaling, the formation of endocytic vesicles, vesicle internalization, nucleocapsid release into the cytoplasm, etc. [11]. AcMNPV enters host cells and mammalian cells by clathrin-mediated endocytosis (CME) and direct membrane fusion (DMF) [12,13], and macropinocytosis plays a key role in AcMNPV entry into mammalian cells [13]. However, the BmNPV entry mechanism is different from that of AcMNPV. BmNPV utilizes macropinocytosis to enter host cells [14], and DMF does not mediate BmNPV infection in host cells [15], which implies that macropinocytosis is an efficient entry pathway for BmNPV. Macropinocytosis is often employed by viruses to expand the host range [16] and is mediated by transient plasma membrane ruffling [16]. Methyl-beta-cyclodextrin (MβCD) can activate membrane ruffling in mammalian cells [17]; coincidentally, MβCD has recently been shown to efficiently enhance BmNPV and AcMNPV infection [18]. Thus, an interesting question is, can MβCD induce membrane ruffling to mediate BmNPV infection in Sf cells?

In this study, we first verified that BmNPV produced a very low-level infection in Sf21 cells; however, MβCD incubation efficiently increased BmNPV infection, which was mediated by the activation of membrane ruffling; inhibitors of macropinocytosis greatly abolished this enhancement. Next, we checked the relationship between the induction time point and infection, and found that incubation before infection produced a better effect than incubation post viral entry. Finally, with the use of an inhibitor, we provide evidence here that BmNPV naturally enters Sf21 cells by the CME pathway, and macropinocytosis is essential for BmNPV infection. Our findings indicate that the activation of macropinocytosis mediates BmNPV infection in nonhost cells, and contributes to the understanding of the BmNPV entry mechanism.

## 2. Materials and Methods 

### 2.1. Cells, Bacmids, and Viruses

BmN [14] (stored in our lab) and Sf21 cells (Thermo Fisher Scientific, Waltham, MA, USA) were cultured at 27 °C in TC-100 insect medium (AppliChem, Darmstadt, Germany) supplemented with 10% fetal bovine serum (Gibco BRL, Gaithersburg, MD, USA) and SF900II SFM (Thermo Fisher Scientific, Waltham, MA, USA) medium, respectively, using standard techniques. The BmBac-ph-egfp bacmid was constructed by inserting a BmNPV *polyhedrin* (ph) gene containing its own promoter and enhanced green fluorescence protein (egfp) controlled by the hsp70 promoter at the *att*TN7 locus of BmBacJS13 [19], according to the instructions in the Bac-to-Bac expression system manual (Thermo Fisher Scientific, Waltham, MA, USA). Bacmid (BmBac-ph-egfp) DNA was transfected into BmN cells using Lipofectamine 2000 (Thermo Fisher Scientific, Waltham, MA, USA). Virus-containing supernatant was collected at 120 h post transfection and used to infect BmN cells for viral amplification. The virus was designated vBmBac-ph-egfp, and the viral titers were determined by end-point dilution assay (EPDA) on BmN cells.

### 2.2. MβCD Treatments and BmNPV Infections

MβCD (Sigma-Aldrich, Saint Louis, MO, USA) stock solutions (100 mM) were prepared in phosphate buffer saline (PBS, pH 7.4). Sf21 cells were seeded in 24-well plates at a density of 1 × 10^5^ cells per well overnight at 27 °C and then incubated with 0.125 mM, 0.25 mM, 0.5 mM, 0.75 mM, 1 mM, 1.5 mM, or 2 mM MβCD (final concentration) for 30 min, while treatment with PBS was used as the control. After drug treatment, wells were washed twice with PBS and then inoculated with vBmBac-ph-egfp at a multiplicity of infection (MOI) of 30 for 2 h. After incubation, the virus-containing medium was removed. The cells were washed again with PBS, and fresh medium was added for routine culture. To detect virus infectivity, the cells were harvested and subjected to flow cytometry (FCM) analysis at 24 h post infection (p.i.). In all experiments, time 0 was defined as the point at which fresh medium was added.

### 2.3. Comparison of Viral One-Step Growth Curves

Sf21 cells (5 × 10^5^ cells) were seeded in six-well plates overnight at 27 °C. Cells were pretreated with/without 0.25 mM MβCD for 30 min at 27 °C and then infected with vBmBac-ph-egfp for 2 h at a MOI of 30. Unbound virus was removed, and cells were washed twice with PBS and cultured in fresh medium. At the appropriate time points (0, 12, 24, 48, 72, and 96 h p.i.), 60 µL of supernatant was collected, and the viral titer was determined by EPDA. Each viral infection was carried out in triplicate. 

### 2.4. Electron Microscopy Analysis

Sf21 cells in flasks (1 × 10^6^ cells) or on glass coverslips in a dish (5 × 10^5^ cells) were incubated with 0.25 mM MβCD or PBS for 30 min; the cells were then infected with vBmBac-ph-egfp at a MOI of 30 for 2 h at 4 °C. The virus-containing medium was removed, and the cells were washed with PBS twice and fixed in 4% glutaraldehyde in PBS for 1 h at room temperature. Sf21 cells on coverslips were subjected to scanning electron microscopy (SEM) and Sf21 cell in flasks were subjected to transmission electron microscopy (TEM) analysis. In another treatment, Sf21 cells incubated with/without 0.25 mM MβCD in flasks and infected with vBmBac-ph-egfp at a MOI of 30 for 2 h at 27 °C were subjected to TEM analysis (72 h p.i.). 

### 2.5. Drug Treatments of Sf21 Cells

Ehop-016 (Ehop), rottlerin (Rot), or latrunculin A (Lat) were prepared in DMSO in accordance with the manufacturer’s recommendations (Sigma-Aldrich, Saint Louis, MO, USA). The concentrations of the inhibitors used to treat cells for the FCM and western blotting analysis were as previously described [14]. Sf21 cells (5 × 10^5^ cells) were seeded in six-well plates overnight and then incubated with inhibitors at the following concentrations: 5 µM Ehop, 5 µM Lat, or 10 µM Rot for 60 min; the control cells were incubated with DMSO. After incubation, the Sf21 cells were treated with 0.25 mM MβCD or PBS (CTRL) for 30 min and infected with vBmBac-ph-egfp at a MOI of 30 for 2 h in the continued presence of the drug. Subsequently, the virus was removed, the cells were washed twice with PBS, and then cultured in SF900IISFM medium. Infectivity was recorded at 72 h p.i. by FCM, Q-PCR, or western blotting.

### 2.6. Incubation Time Points and Infection Assay

The cells were seeded in 24-well plates at a density of 1 × 10^5^ cell per well overnight and were incubated with 0.25 mM MβCD for 30 min and subsequently infected with vBmBac-ph-egfp (pre-MβCD) at a MOI of 30 for 2 h. The unbound virus was removed by washing twice with PBS, and fresh medium was added to the cells for normal culturing at 27 °C. Alternatively, the cells were infected for 2 h, and the unbound virus was removed. Subsequently, the cells were incubated with MβCD (final concentration 0.25 mM) for 1 h (post-MβCD), then the MβCD was removed and replaced with normal culture medium. In the CTRL conditions, cells were infected without any MβCD incubation. The samples were subjected to FCM, Q-PCR, and western blotting at 72 h p.i. 

### 2.7. Analysis of the Endocytic Pathway by Which BmNPV Enters Sf21 Cells

Stock solutions (50 mg/mL) of chlorpromazine (CPZ) (Sigma-Aldrich, Saint Louis, MO, USA) were prepared in water following the manufacturer’s recommendations. Sf21 cells were seeded in 24-well plates at a density of 1 × 10^5^ cell per well overnight and were divided into three groups. The first group of cells (termed no MβCD) were preincubated with increasing doses of CPZ (final concentrations of 0 µg/mL, 25 µg/mL, and 50 µg/mL) for 30 min and then infected with vBmBac-ph-egfp at a MOI of 50 for 2 h in the continued presence of the drug. After removal of the unbound virus and chemical inhibitor, the cells were cultured with fresh medium. The second group of cells (termed pre-MβCD) was preincubated with increasing doses of CPZ and 0.25 mM MβCD for 30 min and subsequently infected with vBmBac-ph-egfp. The remaining group of cells (termed post-MβCD) was first pretreated with increasing doses of CPZ and then infected with vBmBac-ph-egfp. Then, the unbound virus and medium were removed, and the cells were incubated with 0.25 mM MβCD for 1 h. All group samples were subjected to FCM, Q-PCR, and western blotting.

### 2.8. Flow cytometry and Western Blotting

FCM and western blotting were conducted as previously described in [18]. Rabbit anti-VP39 was a gift from Dr. Zhihong Hu (Wuhan Institute of Virology, Chinese Academy of Sciences, China). 

### 2.9. Q-PCR

Total RNA was extracted and subjected to Q-PCR as previously described [20], with primers Lef3F: TGAGCAGTCTGTTGGTGTGA and Lef3R: GCACAGCTTTGAATTGTGCT. GAPDH expression was set as the internal control with primers GAPDH QF: CATTCCGCGTCCCTGTTGCTAAT and GAPDH QR: GCTGCCTCCTTGACCTTTTGC.

### 2.10. Statistical Analysis

All experiments included three independent repeats. The *p* value was calculated using a two-tailed Student’s *t*-test using Microsoft Excel 2016 (Microsoft, Redmond, WA, USA). Asterisks indicate *p* values as follows: * *p* < 0.05, ** *p* < 0.01, and *** *p* < 0.001.

## 3. Results

### 3.1. Incubation with a Low Concentration of MβCD Facilitates BmNPV Infection in Sf21 Cells

**Figure 1:**
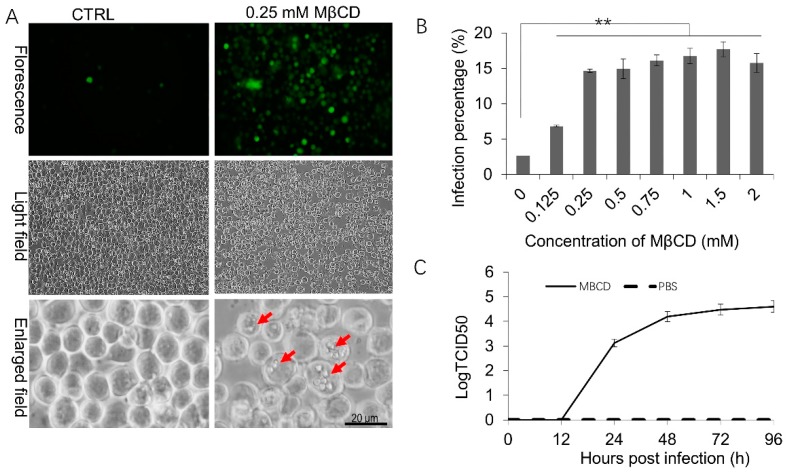
The effects of low-dose methyl-beta-cyclodextrin (MβCD) incubation on BmNPV infection in Sf21 cells. (**A**) Representative images of fluorescence or polyhedrin expression in mock-treated or pretreated Sf21 cells infected by BmNPV. Sf21 cells were pretreated with 0.25 mM MβCD or PBS (CTRL) for 30 min and subsequently infected with vBmBac-ph-egfp for 2 h at a multiplicity of infection (MOI) of 30 and observed at 72 h p.i. Red arrows show the occlusion bodies. (**B**) Flow cytometry analysis of BmNPV infectivity in Sf21 cells in the presence of different concentrations of MβCD. Cells were pretreated with the indicated concentrations of MβCD for 30 min and then infected with vBmBac-ph-egfp (MOI = 30). Viral infectivity was measured at 24 h p.i by analyzing the percentage of cells expressing the reporter gene *egfp*. (** *p* < 0.01). (**C**) Growth curve of BmNPV progeny BV released from Sf21 cells. Similar to (**B**), after cells were treated with 0.25 mM MβCD and infected with vBmBac-ph-egfp at a MOI of 30, the supernatants containing the progeny virus released from the infected Sf21 cells were collected, and the BV titers were determined on BmN cells using the end-point dilution assay method. The experiment was performed in triplicate. The error bars represent the standard deviation.

Sf21 is a nonpermissive cell line for BmNPV in which BmNPV can barely replicate. When we used BmNPV to infect Sf21 cells at a MOI of 30, only a single fluorescent cell was found in the control (CTRL) conditions at 72 h post infection (p.i.) (Figure 1A). However, when the cells were pretreated with 0.25 mM MβCD for 30 min and then infected, neighboring green fluorescent cells were observed (Figure 1A). Occlusion bodies were observed in the late stage of infection in MβCD-treated cells (Figure 1A, red arrow), indicating that MβCD efficiently facilitates BmNPV infection in Sf21 cells. FCM assays were conducted to confirm the enhancement effects of increasing MβCD concentrations on BmNPV infection. When Sf21 cells were preincubated with 0.125 mM, 0.25 mM, 0.5 mM, 0.75 mM, 1 mM, 1.5 mM, and 2 mM MβCD, the infection rates were increased to 6.82%, 14.63%, 14.93%, 16.12%, 16.73%, 17.7%, and 15.76% than the 2.66% of the CTRL (Figure 1B). Significant differences were detected between the MβCD-treated cells and the control cells, which indicates that MβCD incubation increased BmNPV infection in Sf21 cells while AcMNPV infection in Sf cells typically showed over 80–90% infection [12].

Viral replication dynamics are an important characteristic of viral infectivity. Next, one-step growth curve assays were performed to detect progeny virus release from Sf21 cells treated with MβCD. In the presence of 0.25 mM MβCD, the virus showed typical curve dynamics of budding virus (BV) production (Figure 1C). BV titers increased significantly as the infection time increased, and a plateau was achieved from 48 to 96 h p.i. The highest viral titer was achieved at 96 h p.i. with a titer of 3.96 × 10^4^ 50% tissue culture infectious doses. However, the virus that infected the untreated control cell was too low to observe (Figure 1C).

### 3.2. MβCD Incubation Activates Membrane Ruffling, Which Results in BmNPV Infection in Sf21 Cells

**Figure 2:**
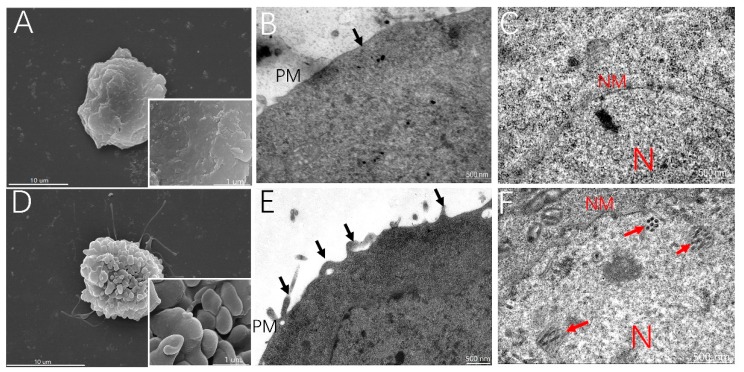
MβCD treatment induced membrane ruffling, facilitating the BmNPV infection of Sf21 cells. Sf21 cells were treated with or without 0.25 mM MβCD for 30 min and infected with vBmBac-ph-egfp at a MOI of 30 for 2 h at 4 °C. The cells were then fixed and processed for electron microscopy. (**A**,**D**) scanning electron microscopy analysis on the surface of cells mock-treated or pretreated with MβCD. (**B**,**E**) transmission electron microscopy analysis of the membrane of cells mock-treated or pretreated with MβCD. Black arrows show the protrusion and closure of ruffles. (**C**,**F**) Internal subcellular analysis of Sf21 cells mock-treated or pretreated with MβCD. Red arrows show the virions. N, nucleus; PM, plasma membrane; NM, nuclear membrane.

Though MβCD incubation increased BmNPV entry in Sf21 cells, the mechanism is not clear. MβCD activates membrane ruffling in mammalian cells [17], so to confirm that MβCD incubation activates membrane ruffling in Sf21 cells, the changes in the morphology of the Sf21 cell surface in the infection was investigated with SEM and TEM. Sf21 cells were preincubated with/without MβCD and infected at 4 °C for 2 h. Then, the cells were fixed and processed for electron microscopy. The SEM results indicated that Sf21 cells without MβCD treatment had a smooth surface (Figure 2A); however, marked membrane ruffling was observed around the MβCD-treated Sf21 cells, and many lamellipodium-like protrusions were formed on the cell surface (Figure 2D). The ultrathin section structure also showed that the cells without MβCD incubation had a smooth surface (Figure 2B), while incubation with 0.25 mM MβCD prior to infection resulted in vigorous membrane ruffling and closure at the cell surface (Figure 2E, arrows). Moreover, some viral nucleocapsids were found at 72 h p.i. in the nuclei of cells treated with MβCD (Figure 2F, white arrows), and a typical cytopathic effect of mitochondria was also observed, which was difficult to find in cells not incubated with MβCD (Figure 2C). These results indicate that preincubation with 0.25 mM MβCD prior to infection activates membrane ruffling of Sf21 cells and facilitates BmNPV infection of Sf21 cells.

### 3.3. Inhibition of Membrane Ruffling Greatly Reduces the MβCD-Induced Increase in BmNPV Infection of Sf21 Cells

**Figure 3:**
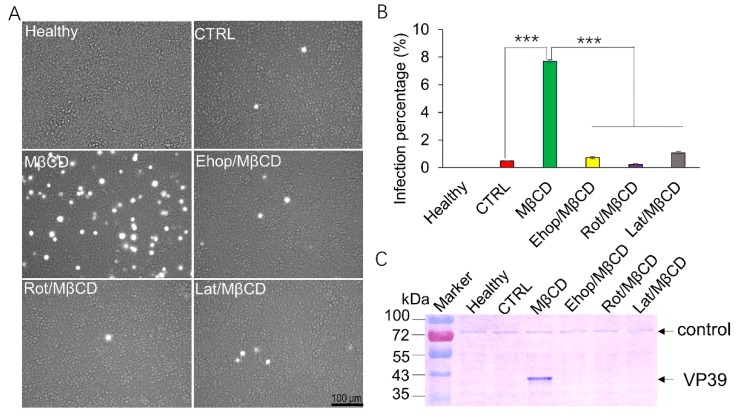
Effect of macropinocytosis inhibitor treatment on BmNPV infection in Sf21 cells. Sf21 cells were mock-treated or pretreated with different inhibitors (Ehop, Rot, or Lat) for 60 min, then treated with 0.25 mM MβCD or PBS (CTRL) for 30 min and subsequently infected with vBmBac-ph-egfp for 2 h at a MOI of 30. (**A**) Representative images of fluorescence expression in Sf21 cells infected with BmNPV at 72 h p.i. Scale bars, 100 µm. (**B**) Flow cytometry analysis of BmNPV infection in Sf21 cells. (*** *p* < 0.001 by Student’s *t*-test). (**C**) Expression of BmNPV VP39 in Sf21 cells by western blotting using an anti-VP39 primary antibody.

Membrane ruffling is dependent on the organization of filamentous actin. Rac1 [21] and protein kinase C (PKC) [22] are responsible for triggering membrane ruffles and macropinosome closure [23]. To further investigate whether membrane ruffling increased BmNPV entry into Sf21 cells, Ehop, Rot, or Lat, which are inhibitors of Rac1, PKC kinases, and actin polymerization, respectively, were applied during the infection. Sf21 cells were mock-treated or pretreated with Ehop, Rot, and Lat for 60 min; then, the cells were induced with 0.25 mM MβCD and infected. As expected, the infection percentage induced by MβCD was effectively reduced by inhibitor treatment (Figure 3A). When the cells were pretreated with Ehop/MβCD, Rot/MβCD, and Lat/MβCD, there was no obvious increase in EGFP expression in the cells and was similar to that of the CTRL, while many of the cells incubated with MβCD alone exhibited green fluorescence. The viral infection efficiency was recorded by FCM, which showed that MβCD treatment alone increased the infection rate by 7.9% from the CTRL rate of 0.48%. When the cells were pretreated with 5 µM Ehop, 10 µM Rot, or 5 µM Lat then treated with MβCD, the infection rate was decreased to 0.71%, 0.21%, and 1.05%, respectively. Significant differences were detected between MβCD treatment, drug/MβCD treatments, and the CTRL treatment (Figure 3B). In addition, expression of the late protein VP39 was used to further verify viral infections by western blotting, as shown in Figure 3C. An unspecific control band was stably expressed in all samples, while significant VP39 protein synthesis was observed in the cells pretreated with MβCD alone (Figure 3C). There was an almost nonspecific VP39 immunoreactive band detected in the healthy, CTRL, and inhibitor pretreated cells. These results indicate that inhibition of membrane ruffling can greatly abolish BmNPV infection in Sf21 cells.

### 3.4. MβCD Incubation Post Viral Entry Increases BmNPV Infection of Sf21 Cells

**Figure 4:**
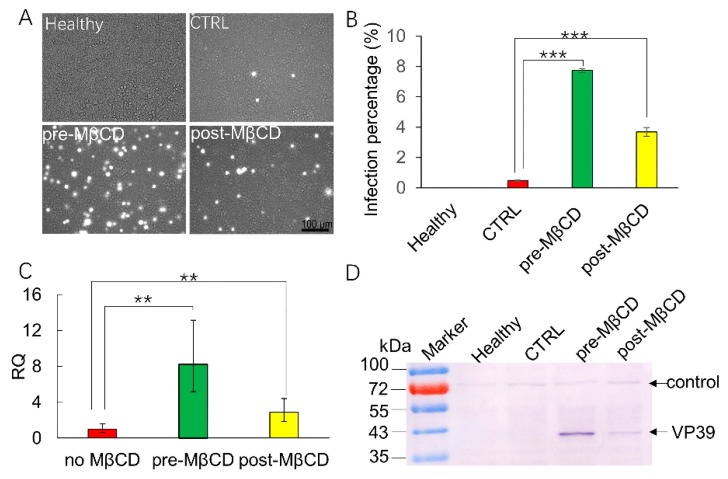
MβCD induction post infection rescues BmNPV infection in Sf21 cells. Sf21 cells were mock-treated, or MβCD-treated (0.25 mM) for 30 min before or after vBmBac-ph-egfp infection for 2 h (MOI = 30). (**A**) Representative images of fluorescence expression in Sf21 cells infected with vBmBac-ph-egfp at 72 h p.i. Scale bars, 100 µm. (**B**) Flow cytometry analysis of vBmBac-ph-egfp infection in Sf21 cells at 72 h p.i. (** *p* < 0.01, *** *p* < 0.001 by the Student’s *t*-test). (**C**) Relative transcription of *lef-3* in the infected Sf21 cells by Q-PCR. (**D**) VP39 expression was detected using an anti-VP39 antibody.

Membrane ruffling can facilitate the engulfment of virions in the media directly by macropinocytic endocytosis; in addition, some virions can be internalized by clathrin-mediated endocytosis, and the induction of macropinocytosis in parallel assists their infection. To further verify the role of macropinocytosis in BmNPV entry into Sf21 cells, cells were treated with MβCD before (pre-MβCD) or after (post-MβCD) viral entry. Fluorescence images showed that only a few fluorescent cells were present in the CTRL conditions, while many fluorescent cells were found in the pre-MβCD conditions. Interestingly, more fluorescent cells were observed in the post-MβCD treatment group than in the CTRL group (Figure 4A), with a significantly increased infection rate of 3.68% when compared to the CTRL infection rate of 0.45% by the FCM assay, however, the post-MβCD infection rate was lower than the pre-MβCD infection rate of 7.6% (Figure 4B). *Lef-3*, a late transcribed gene, was selected for Q-PCR validation, the relative quantity (RQ) of the CTRL group was set as 1; accordingly, the relative expression in the pre-MβCD and post-MβCD groups was increased by 8.24- and 2.86-fold, respectively (Figure 4C). A significant difference was detected between these treatments. VP39 expression revealed similar trends between the CTRL and pre/post-MβCD treatments (Figure 4D). These results imply that the induction of macropinocytosis post BmNPV internalization also increased the infection of Sf21 cells. For the low level of infection in CTRL conditions without MβCD incubation, we suggest that the induction of macropinocytosis assists BmNPV infection, and BmNPV enters Sf21 cells through another pathway.

### 3.5. BmNPV Entry into Sf21 Cells Via CME

**Figure 5:**
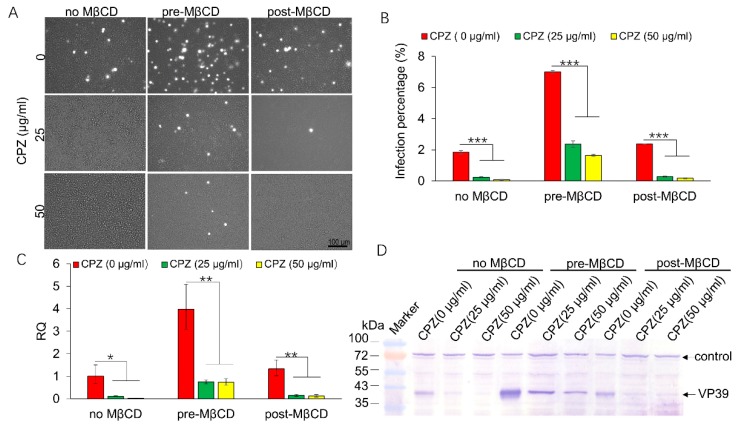
Effects of CPZ on BmNPV infection in Sf21 cells. Sf21 cells were treated with CPZ for 30 min and then infected with vBmBac-ph-egfp at a MOI of 50 for 2 h in the mock-treated group or pretreated with 0.25 MβCD for 1 h (no MβCD, pre-MβCD) or post-treated with MβCD after virus infection (post-MβCD). At 72 h p.i., the cells were harvested. (**A**) Representative images of fluorescence expression in Sf21 cells infected with BmNPV. Bars, 100 µm. (**B**) Infection percentage of cells treated with CPZ and MβCD assessed by FCM. (**C**) Relative transcription of *lef-3* in CPZ- and MβCD-treated cells. The gene transcription in the 0 µg/mL MβCD treatment condition was set as 1. (**D**) VP39 expression in Sf21 cells treated with increasing concentrations of CPZ in the presence or absence of MβCD using an anti-BmNPV VP39 primary antibody. * *p* < 0.05, ** *p* < 0.01, *** *p* < 0.001 by the Student’s *t*-test.

Viral utilization of the endocytic pathway is cell-type dependent [24], and AcMNPV enters Sf9 cells by CME [12]. Next, we examined whether BmNPV enters Sf21 cells by the CME mechanism. We selected chlorpromazine to inhibit CME, as it is widely used to block CME by preventing the assembly of clathrin-coated pits at the plasma membrane [25]. Our result showed that CPZ treatment efficiently inhibited viral infection. As shown in Figure 5A, 1.85% of Sf21 cells exhibited green fluorescence in the CTRL conditions without CPZ treatment, while in the treatments with 25 µg/mL and 50 µg/mL CPZ, only 0.24% and 0.086% of cells were infected (Figure 5B, no MβCD), and the infection rate was significantly decreased to ~12.9% and 4.6%, respectively. When cells were preincubated with CPZ and MβCD, the infection percentages decreased from 7.00% to 2.36% and 1.65% in 0, 25, and 50 µg/mL CPZ-treated cells, respectively (Figure 5B pre-MβCD), and the infection rate was significantly decreased to ~33.7% and 23.6%. Furthermore, when the cells were preincubated with CPZ and then incubated with MβCD after viral entry, 2.37%, 0.28%, and 0.18% of Sf21 cells were infected in the 0, 25, and 50 µg/mL CPZ-treatment conditions, respectively (Figure 5B post-MβCD), the infection rate was significantly decreased to ~11.8% and 7.6%, and significant differences were detected between the CPZ treatments in the three groups. Q-PCR also verified these significant differences. As shown in Figure 5C, the relative transcription of *lef-3* in the 25 and 50 µg/mL CPZ-treated cells was only 11% and 1.8% greater than 100% of the cells without CPZ treatment. In the pre-MβCD treatment conditions, only 18.7% and 18.4% of the transcription was retained in cells treated with the two concentrations of CPZ. When the cells were incubated with MβCD post infection, the infection rate increased by 36%; however, only 10.8% and 0.85% relative *lef-3* transcription was detected in cells treated with CPZ at the two concentrations, and these results were in accordance with that of FCM assay. The expression of VP39 verified a similar effect of CPZ on BmNPV infection in Sf21 cells; its expression decreased as the CPZ treatment concentration increased (Figure 5D, lower panel arrow), while the unspecific control band was stably expressed in all treatments (Figure 5D, upper panel arrow). These results show that CPZ efficiently inhibited BmNPV infection, indicating that BmNPV mainly utilizes CME to enter Sf21 cells.

## 4. Discussion

The BmNPV entry mechanism is different from that of AcMNPV as BmNPV enters host cells by macropinocytosis [14], and DMF results in an abortive infection in BmNPV-infected cells [15], which indicates that macropinocytosis is an efficient entry pathway for BmNPV entry. BmNPV infection is dependent on membrane cholesterol, and a high concentration MβCD inhibits its infection [14]. However, low concentration MβCD enhanced its infection [18], which resulted from membrane ruffling being activated. Here, we show that MβCD-induced membrane ruffling mediates BmNPV infection in nonpermissive cells. The application of membrane ruffling inhibitors efficiently abolished this enhancement. Though a single inhibitor has a broad effect, the application of more inhibitors can help complement our data. This further indicates that macropinocytosis is involved in BmNPV infection in nonhost cells. However, macropinocytosis plays differential roles in virus infection. First, it is the key entry pathway for direct virion engulfment in the infection process of viruses such as the vaccinia virus [16] and African swine fever virus [26]. Second, the induction of macropinocytosis assists the entry and infectivity of some viruses that are internalized by other endocytosis pathways such as Ad2, which induces macropinocytosis in parallel for subsequent penetration; the lysed macropinosome contents are required for its infection [27]. Thus, the induction time point of macropinocytosis is important for efficient viral infection. Our results indicate that preincubation with MβCD produced an increased infection percentage (Figure 3, Figure 4 and Figure 5). After viral internalization had been completed, treatment of the cells with MβCD also enhanced the infection, which indicates that the induction of macropinocytosis assists BmNPV entry, and the lysed macropinosome contents may be required for BmNPV infection. However, the increased infection rate in post-MβCD was lower than that of cells preincubated with MβCD (Figure 4 and Figure 5). These results imply that synchronization of macropinocytosis induction and viral internalization is important for productive BmNPV infections in Sf21 cells. 

The mechanism of BmNPV entry into Sf21 cells is difficult to verify by inhibitor application due to its very low infection rate; however, when we increased the MOI of BmNPV, this low-level replication was detected in Sf21 cells. In addition, we show here that MβCD incubation efficiently increased the infection, thus providing a method to identify the entry pathway by MβCD-induced magnification of the infection. The infection rate of Sf21 cells pretreated with 25 µg/mL CPZ in no- or post-MβCD treatments was significantly decreased to ~12% by FCM and Q-PCR, which indicates that 25 µg/mL CPZ can inhibit BmNPV entry with an efficiency of about ~88%. Furthermore, the infection rate in the pre-MβCD group decreased to ~67% only in the 25 µg/mL CPZ-treated cells, which suggests that the additional ~21% infection may result from the activated macropinocytosis. The preincubation-induced membrane ruffling engulfs BmNPV virions in the media directly, but this engulfment mechanism is less efficient [16] than CME as it is a rapid process with surface-bound virions entering within minutes after attachment [24], so BmNPV mainly enters Sf21 cells via the CME pathway (over 90%). Taken together, our results suggest that BmNPV enters into Sf21 cells mainly via CME, but parallel macropinocytosis enhances the ability of BmNPV to infect the cells once the virus gets in via CME.

The Sf9 cell line is derived from the Sf21 cell line [28], and both AcMNPV and BmNPV enter the Sf cells by CME; therefore, these studies suggest that the Sf cell membrane has a specific receptor to activate the CME pathway. In AcMNPV infections, the nucleocapsid can be transported into the nucleus [29] via interaction with actin/P78-83 [12,30]. However, BmNPV nucleocapsid transportation is not accomplished in the host cell by DMF, which indicates that macropinocytosis is essential for BmNPV to bypass this obstacle [15]. Consistent with the BmNPV infection outcome in Sf9 cells [4], BmNPV produces an abortive infection in Sf21 cells, however, MβCD-induced membrane ruffling partially rescues this failure, further indicating that macropinocytosis is essential for BmNPV infection. In macropinocytic endocytosis, macropinosomes move deep into the cytoplasm [31] and fuse with endolysosomes/lysosomes; most internalized virions are degraded in endolysosomes/lysosomes [32], which mediate virus production during infection [33], and in some cases, the lysed macropinosomes, which are induced simultaneously by viral entry by another pathway, assist some viruses in penetrating and infecting cells [16,27]. Thus, MβCD-induced macropinocytosis may assist BmNPV virions penetrating and thus rescue BmNPV infection in Sf21 cells by CME internalization.

Though BmNPV can replicate in Sf21 cells with the aid of MβCD, the replication rate is still lower than that in BmN cells, which indicates that the entry mechanism may be only one of the host determinants. Several genes, which allow the virus to replicate in certain cells, have been reported as host range factor determinants. A notable factor is host cell-specific factor-1 (*hcf-1*), which is absent in the BmNPV genome [3]. *Hcf-1* is required for productive AcMNPV infections in nonpermissive Tn368 cells [34,35]; HCF-1 is an early protein that localizes to the cell nucleus [36], and promotes the replication of recombinant BmNPV in nonpermissive Tn368 cells [37]. In addition, GP64 has been identified as an AcMNPV and BmNPV host range determinant [1] and AcMNPV GP64 mediates BmNPV replication in Sf9 cells [4], thus, further work is needed to improve BmNPV infections in nonpermissive cells. 

## 5. Conclusions

In conclusion, we demonstrated that BmNPV enters Sf21 cells via CME, resulting in a very low replication rate. Incubation of cells with a low concentration of MβCD, either preincubation or post incubation activates macropinocytosis in Sf21 cells, which mediates BmNPV replication in Sf21 cells. Furthermore, the macropinocytosis pathway inhibitor treatment of cells greatly abolished this enhancement. These results suggest that macropinocytosis mediates BmNPV infection in nonhost cells without any genetic modification to the viral genome.
